# Evaluation of toxicity and anti-amylase activity of 7, 8 dihydroxy coumarin (Daphnetin), a novel α-amylase blocker *in vitro* and *in vivo*

**DOI:** 10.1016/j.toxrep.2025.101991

**Published:** 2025-03-15

**Authors:** Sayan Kar, Sagnik Dutta, Shraddha Saha, Kasturi Sarkar, Shreya Chatterjee, Nabanita Giri, Parames C. Sil

**Affiliations:** a Department of Microbiology St. Xavier’s College, Kolkata, India; b Department of Microbiology, University of Calcutta, India; c Department of Physiology, University of Calcutta, India; d Department of Microbiology Acharya Prafulla Chandra College, India; e Department of Molecular medicine, Bose Institute, India

**Keywords:** Dihydroxy coumarin, Anti-amylase property, Post-prandial blood glucose, Oral starch tolerance test

## Abstract

Management of postprandial blood glucose is a measure in regulating the blood sugar concentration. This paper evaluates the anti-amylase property of a natural compound 7,8 dihydroxycoumarin (daphnetin) both *in vitro* and *in vivo*. The inhibitory effect of daphnetin was evaluated against salivary (HSA) and pancreatic α-amylases (PPA) and α-glucosidase (AG) *in vitro*. Enzyme kinetics studies revealed that the inhibition of HSA, PPA and AG by daphnetin was competitive in nature. Pretreatment with daphnetin was found to inhibit the amylases *in vivo* as confirmed by the oral starch tolerance test (OSTT). No adverse effect of daphnetin was observed on serum markers and organs like liver, kidney and small intestine. The impact of the inhibitory effect of daphnetin in *in vivo* was comparable to the positive control, acarbose in all aspects.

## Highlights

• 7,8 dihydroxycoumarin or Daphnetin inhibits α-amylases and α-glucosidase *in vitro*.
• The inhibition of the enzymes by daphnetin was competitive in nature.
• Oral starch tolerance test confirmed anti-amylase property of daphnetin *in vivo.*
• Administration of daphnetin for 7 days had no adverse effect on the mice.
• The results were comparable to acarbose, the positive control.

## Graphical Abstract

**Figure:**
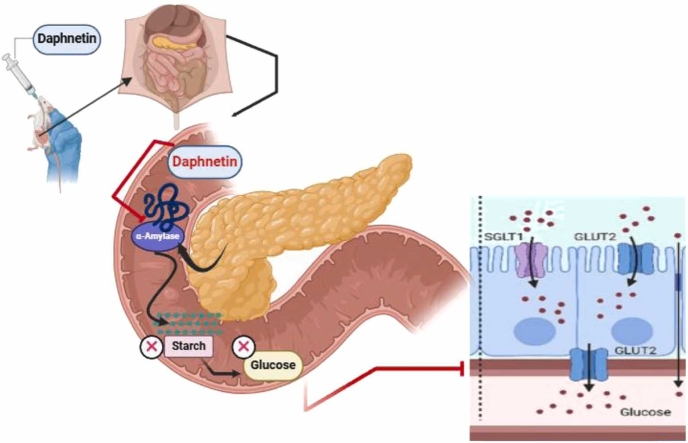

## 1 Introduction

Diabetes mellitus type 2 can be effectively controlled by managing the postprandial (PP) blood sugar level [1], [2]. Postprandial glycemia is responsible and associated to cardiovascular complications and a risk factor for atherosclerosis [3], [4], [5]. Elevation of postprandial plasma glucose becomes the evidence of metabolic abnormalities like improper functioning of β-cells and insulin secretion [6], [7].

Hence, reduction or management of PP sugar level can reduce the incidence and severity of long-term effects of diabetes [3]. Other than standard measures like insulin injections and medications that control glucagon-insulin balance in blood, PP blood glucose concentration can also be controlled by preventing the activity of α-amylases and glucosidases in saliva and in the gut. α-amylase, which hydrolyses α-D1–4 glycosidic linkages in starch and related polysaccharides, belongs to the glycoside hydrolase family 13. There are α-amylase blockers available in the market which include acarbose, miglitol, voglibose etc. As these molecules have considerable side effects like abdominal discomfort, diarrhoea etc, the current focus is on plant-based molecules having anti α-amylase properties with minimal harmful effects. Several plant derivatives are known to have antidiabetic [8], [9], hepatoprotective [10], [11], [12], anticancer properties [13], [14], antioxidant [15], [16], [17], antibacterial [14] etc.

In search of novel α-amylase blockers, we have screened several dihydroxycoumarins (DHCs), which are secondary metabolites synthesized by plants like *Coumarouna odorata*, *Anthoxanthum odoratum*, *Cinnamomum cassia*, cherry blossom trees etc [18], [19]. The DHCs are known for properties like anti-inflammatory, anticancer, anticoagulant, antibacterial, antiviral, anticonvulsant, anti-hypertensive, anti-hyperglycemic etc. [20], [21], [22], [23]. After *in silico* screening and *in vitro* confirmation, we have identified a molecule, 7,8 dihydroxycoumarin or daphnetin which potentially inhibits both salivary and pancreatic α-amylase and α-glucosidase *in vitro*. The anti-amylase property of this molecule was confirmed *in vivo* in a mice model. No increase in blood sugar level was observed after oral starch tolerance test when pretreated with daphnetin. Daphnetin, which is an immunosuppressive and a protein kinase, is found in many plants including Daphne Korean Nakai [24]. This molecule is reported to kill multidrug-resistant *H. pylori* by increasing DNA damage and altering its membrane structure. Daphnetin also prevents the attachment of *H. pylori* to human gastric epithelial cells (GES-1 cells) [25].

In this study, we have discussed the effect of daphnetin on three important enzymes required to breakdown starch in our GI tract viz. salivary α-amylase, pancreatic α-amylase and α-glucosidase. Also, we have focussed on evaluating the pathophysiological effect of daphnetin through serum markers and histopathological studies.

## 2 Materials and methods

### 2.1 Chemicals and reagents

7,8 Dihydroxycoumarin (daphnetin), acarbose, human salivary α amylase (HSA), porcine pancreatic amylase (PPA) and α-glucosidase (AG) from Sigma-Aldrich. Other chemicals were obtained from Hi-Media. India.

### 2.2 α-amylase and α-glucosidase inhibition assay in *in vitro* condition

HSA, PPA and AG inhibition assay was performed according to the standard DNSA method [26]. Equal volumes of enzyme (0.05 mg/ml) and inhibitor (1 mg/ml) were mixed and incubated for 10 min (min) at 25 °C. Same volume of starch (0.2 mg/ml) was added and incubated for another 5 mins. Next, double volume of DNSA was added and incubated in a water bath at 90 °C for 5 mins. The reaction was stopped by the addition of sodium potassium tartrate solution and OD was taken at 540 nm after cooling. The percentage of inhibition was measured by the following formula:

Percentage inhibition =

(Absorbance without inhibitor –Absorbance with inhibitor) / Absorbance without inhibitor

The velocities of the reactions were determined in each case. Acarbose was used as the positive control.

### 2.3 Determination of the inhibitory concentration (IC50) values of daphnetin and acarbose

The IC50 values of daphnetin, and acarbose were determined against HSA, PPA and AG from plots of % inhibition versus inhibitor concentration and calculated by logarithmic regression analysis [27]. Six different concentrations (0.005, 0.01, 0.02, 0.025, 0.05 and 0.08 mg/ml) were used for both the molecules, keeping all other parameters constant. The experiments were replicated and measured against suitable controls every time.

### 2.4 Determination of the nature of enzyme inhibition

The nature of inhibition of HSA, PPA and AG by daphnetin, and acarbose was determined from the 1/v vs 1/s plot. Here the concentration of starch was varied (0.2, 0.4, 0.6, 0.8, 1 mg/ml), keeping enzyme and inhibitor concentration the same. For each concentration of starch, the velocity of the reaction was determined from the amount of glucose produced. The Lineweaver Burk plots for kinetic analysis were drawn for each inhibitor against HSA, PPA and AG [28].

### 2.5 Ligand binding studies through fluorescence measurement

Interaction between HSA and daphnetin was studied by measuring the changes in intrinsic fluorescence intensity [29]. Titration of HSA with different concentrations of daphnetin (0.0003, 0.0006 and 0.0012 M) was monitored by following the changes in fluorescence at 350 nm and was compared with the fluorescence intensity of HSA alone. Important thermodynamic parameters like association constants (K_a_), Gibbs free energy changes (ΔG°) of binding for daphnetin were determined at various temperatures; 301 K, 310 K and 323 K from the fluorescence quenching studies.

### 2.6 Experimental model

Male Swiss albino mice of 6–10 weeks and average weight 20–28 g were acclimatized for 7 days following the approval of the animal ethics committee. The animals were maintained under standard conditions (12hrs light and dark cycle) and were allowed free access to a balanced diet and water ad libitum. The animals were divided into three groups each consisting of 6–8 mice: group 1- normal control; group 2- daphnetin treated; group 3- acarbose treated (positive control).

### 2.7 Oral starch tolerance test (OSTT)

The oral starch tolerance test measures the body's response to starch breakdown by α-amylases in the gut, followed by absorption of glucose into blood.

Fasting blood sugar was measured in all the mice. On the next day, the mice were given starch orally at a concentration of 5 gm/kg body weight [30]. After 30 mins of starch administration, blood glucose concentrations were measured after every 30 mins for 2hrs.

The dose of acarbose initially used was according to the report of Zhang et al. [30]. However, the dose was modified and standardized according to our experimental animals and requirements. The modified dose of daphnetin/acarbose applied was 1.5 mg/kg body weight of mouse 10 mins prior to OSTT. After 10 mins, the mice received starch at the above-mentioned dose (5 gm/kg body weight). Blood glucose concentrations were measured after every 30 mins for 2hrs by puncturing the tail veins. The two values of blood glucose concentrations were compared in each mouse with and without daphnetin/acarbose following OSTT. The blood glucose concentrations were plotted against time for all the mice in each group.

Several doses of daphnetin were checked for its effectivity. The dose of 2 mg/kg body weight was found to be lethal whereas a dose of 1 mg/kg body weight had less inhibitory activity against α-amylase. Hence, an intermediary dose of 1.5 mg/kg body weight was chosen for the experiment and found to work properly.

### 2.8 Toxicity evaluation

For evaluation of toxicity, the mice were given daphnetin at a dose of 1.5 mg/kg body once daily for 7 consecutive days. After sacrificing the mice, organs like liver, kidney, small intestine were collected, and histopathological slides were prepared [31].

### 2.9 Effect of daphnetin on sex-dependent differences in mice

It is known that sex of an animal (male or female) influences carbohydrate metabolism. Also, metabolic disorders vary among male and female animals [32]. The expression of AMY1 gene, which codes for α-amylase 1 varies greatly along different parts of the gut and in male and female organisms [33]. Hence, we tried to evaluate the anti-amylase activity of daphnetin on the post prandial blood glucose level in both the sexes of mice. There were no significant changes of the anti-amylase activity of daphnetin in male and female mice as observed during the treatment. Hence all the data presented in this paper were collected from male mice.

### 2.10 Assessment of the serum markers

#### 2.10.1 ALP Assay

Alkaline phosphatase (ALP) assay was performed following the standard protocol. 20 µl of serum samples were mixed with 1 ml of reagent containing 4-nitrophenyl phosphate (4-NPP). 4-NPP was converted to 4-nitrophenol, having intense yellow colour and absorbance between 405 and 415 nm. The intensity of the colour is proportional to the activity of ALP present in the sample [34].

#### 2.10.2 LDH assay

LDH assay was performed following standard protocol. 20 µl of serum sample was mixed with 1 ml of reaction mixture containing pyruvate and NADH and incubated at RT for 1 min. The rate of oxidation of NADH to NAD+ was measured at 405 nm, which was proportional to the LDH activity in the sample. [35]

### 2.11 Tissue preparation and Hematoxylin and Eosin (H&E) staining

To monitor the cellular structure, shape, pattern and cytotoxic determinants, liver, intestine and kidney tissues were collected properly from all the three groups. The entire tissue preparation process involved four significant steps e.g, fixation, dehydration, clearing and wax infiltration. After fixation in 10 % formalin, graded dehydration was done in 50 %, 70 %, 90 % and 100 % ethanol to eliminate the water content in the tissues [36]. All the samples were then embedded in paraffin wax (Merck 58–60°C paraffin wax white) and the precise tissue blocks were prepared respectively. The blocks were sectioned at 4–5μm with the help of a standard rotary microtome [37].

The H&E staining was performed rigorously on all the selected sectioned tissues followed by the process of deparaffinization through xylene. The overall staining procedure involved deparaffinization, dehydration, addition of dye and tissue mounting. DPX, a mixture of distyrene, a plasticiser, and xylene was used as the mounting agent [38].

### 2.12 1,1-diphenyl-1-picrylhydrazyl (DPPH) scavenging activity

Many plant extracts are known to possess antioxidant properties [39], [40], [41]. The free radical scavenging activity of daphnetin was determined using a stable radical 1,1-diphenyl-2-picrylhydrazyl (DPPH). 2.5 mg of DPPH was dissolved in 100 ml of 95 % ethanol and 3.9 ml of freshly prepared DPPH was mixed with 100 µl of daphnetin of different concentrations and incubated for 30 mins in room temperature. Absorbance was measured at 517 nm. The capacity to scavenge the DPPH radical was calculated, using the following equation:

DPPH scavenged (%) = {(Ac – At) / Ac} x 100

Where Ac is the absorbance of the control reaction and At is the absorbance of the sample

reactions. An antioxidant value of 100 % indicates the strongest antioxidant activity and 95 %

ethanol as blank and the DPPH – ethanol mixture as control was used [39].

### 2.13 Statistical analysis

All experiments were replicated at least thrice. Means, standard errors, and standard deviations (SD) were calculated from replicates within the experiments. Statistical analysis was done by one way analysis of variance (ANOVA). Statistical significance was accepted at *P* < 0.05. IC50 was calculated using graph pad prism software.

## 3 Results

### 3.1 Inhibitory activity of daphnetin against α-amylase and α-glucosidase

Daphnetin showed potent anti HSA, PPA and AG activity of 90 %, 100 % and 52 % compared to 89 %, 40 % and 95 % inhibitory activity of acarbose against the respective enzymes at the same concentration *in vitro* (Table 1).

**Table 1:** Percent inhibition and nature of inhibition of HSA, PPA and AG by daphnetin and acarbose.

	Nature and % of inhibition	Km	Vmax
Daph against HSA	Competitive, ∼89 %	0.267 mg/ml (- I) 1.67 mg/ml (+I)	0.03 mg/ml/min
Daph against PPA	Competitive, ∼100 %	0.235 mg/ml (-I) 0.364 mg/ml (+I)	0.05 mg/ml/min
Daph against AG	Competitive, ∼52 %	0.2 mg/ml (-I) 0.8 mg/ml (+I)	0.05 mg/ml/min
Acarbose against HSA	Competitive, ∼90 %	0.25 mg/ml (-I) 0.8 mg/ml (+I)	0.033 mg/ml/min
Acarbose against PPA	Non-competitive, ∼40 %	0.11 mg/ml	0.025 mg/ml/min (+I) 0.03 mg/ml/min (-I)
Acarbose against AG	Competitive, ∼95 %	0.286 mg/ml (-I) 0.8 mg/ml (+I)	0.033 mg/ml/min

### 3.2 Determination of IC50 values

The IC50 values of daphnetin were 0.98 ± 0.08 mg/ml, 0.080+ 0.02 mg/ml and 0.035+ 0.01 mg/ml against HSA, PPA and AG respectively compared to the values of 0.05 ± 0.03 mg/ml, 0.03+ 0.03 mg/ml and 0.09+ 0.04 mg/ml of acarbose. (Table 2).

**Table 2:** IC50 values of daphnetin and acarbose against HSA, PPA and AG.

	IC50 value (Daphnetin)	IC50 value (Acarbose)
Salivary amylase	0.98 ± 0.08 mg/ml	0.05 ± 0.03 mg/ml
Pancreatic amylase	0.080+ 0.02 mg/ml	0.03+ 0.03 mg/ml
Alpha-glucosidase	0.035+ 0.01 mg/ml	0.09+ 0.04 mg/ml

### 3.3 Nature of inhibition

**Fig. 1:**
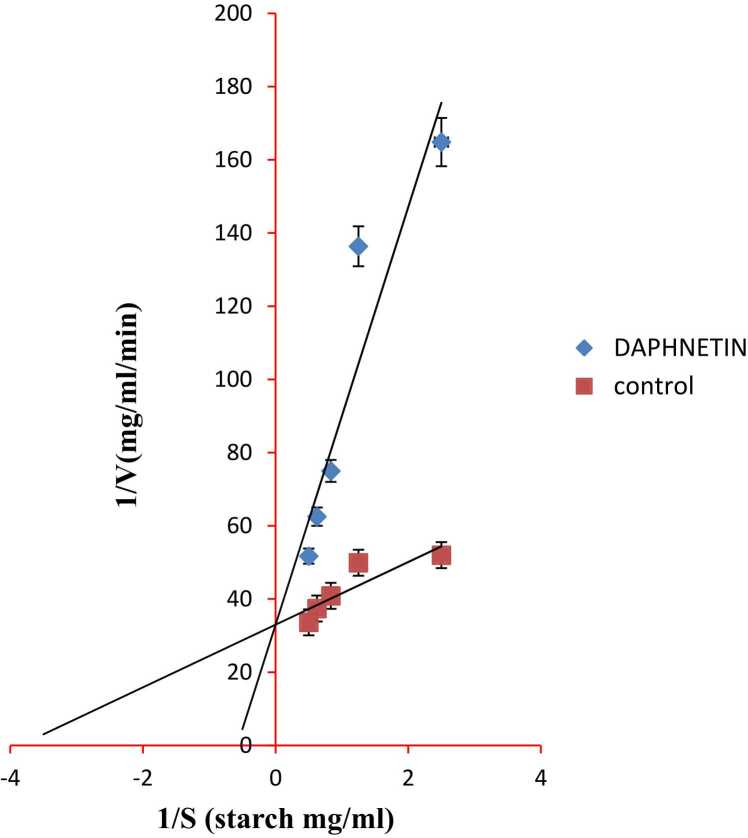
Lineweaver–Burk plots for inhibition of HSA by daphnetin. Error bars represent mean± SD of triplicates and p values < 0.05 were considered significant.

**Fig. 2:**
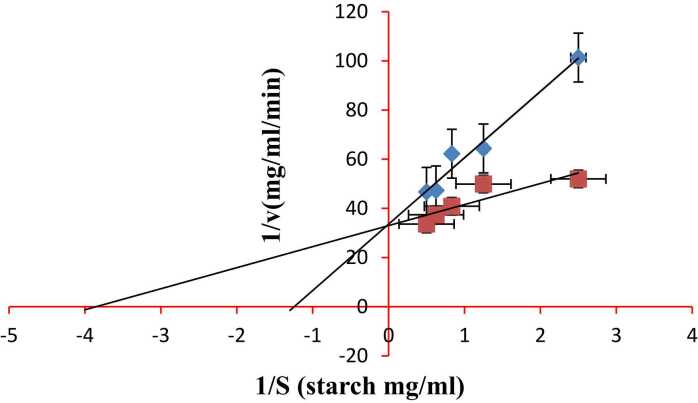
Lineweaver–Burk plots for inhibition of HSA by acarbose. Error bars represent mean± SD of triplicates and p values < 0.05 were considered significant.

Daphnetin showed a competitive mode of inhibition against HSA, PPA and AG *in vitro*. The Vmax value, 0.03 mg/ml/min remains unchanged with and without daphnetin against HSA while Km changes from 0.267 mg/ml without inhibitor to 1.67 mg/ml with inhibitor (Fig. 1). Acarbose showed a competitive mode of inhibition against HSA with changes in Km (Fig. 2 and Table 1). The Vmax value (0.05 mg/ml/min) remained unchanged with and without daphnetin against PPA while Km value changed from 0.235 mg/ml without inhibitor to 0.364 mg/ml with inhibitor (Figure no shown, data communicated). The Vmax value, 0.05 mg/ml/min remains unchanged with and without daphnetin against AG while Km value changes from 0.2 mg/ml without inhibitor to 0.8 mg/ml with inhibitor (Figure no shown, data communicated). Acarbose showed competitive mode of inhibition against AG and non-competitive mode of inhibition against PPA. (Table 1)

### 3.4 Ligand binding studies

**Fig. 3:**
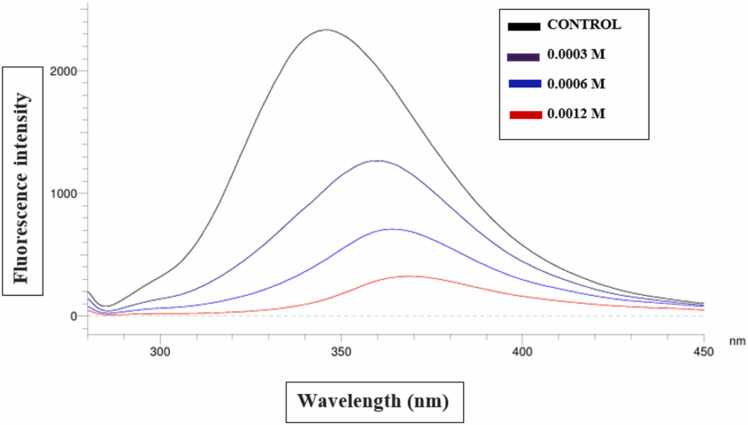

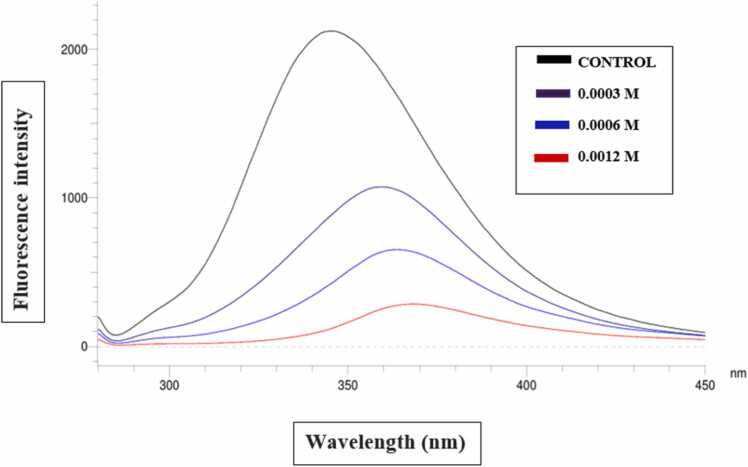
Intrinsic fluorescence spectra of HSA at different concentrations of daphnetin (0–0.0012 M) at three different temperatures. (A) 28 °C or 301 K, (B) 37 °C or 310 K, and (C) 50 °C or 323 K.

Fluorescence quenching of HSA with daphnetin was studied to gain insight into the mechanism of ligand interaction. The interaction between daphnetin and HSA was followed by the changes in the fluorescence emission spectra of HSA in the absence and presence of the inhibitor. Fig. 3 shows the fluorescence emission spectra of HSA at pH 6.8 and temperature 301 K with daphnetin at a concentration range of 0–0.0012 M. The fluorescence peak at 345 nm quenches and shifts towards higher wavelengths (red shift) as observed with increase in the concentrations of daphnetin. The possible explanation for this observation might be that daphnetin binding to HSA results in the unfolding of the fluorescent amino acid residues that cause quenching of its inherent fluorescence properties. Using docking studies, it has been seen that daphnetin binds to the tyrosine 2 residue of HSA [figure not shown]. Fluorescence quenching of HSA by daphnetin had been repeated at higher temperatures, 310 K and 323 K and F0/F vs conc. of inhibitor was plotted to find out the Stern Volmer constant (Ksv). Calculation of Stern-Volmer quenching constant by linear regression analysis for daphnetin was found to remain almost constant (6.01 ×10^3^ M^−1^, 6.19 ×10^3^ M^−1^, 6.19 ×10^3^ M^−1^ with R2 values of 0.9954, 0.9885 and 0.9885, at temp 301 K, 310 K and 323 K respectively) (Fig. 3). The ΔG° value calculated from the binding constant is −21.55 kJ /mol at 301 K, indicating favourable interactions taking place between HSA and daphnetin.

### 3.5 Oral starch tolerance test

**Fig. 4:**
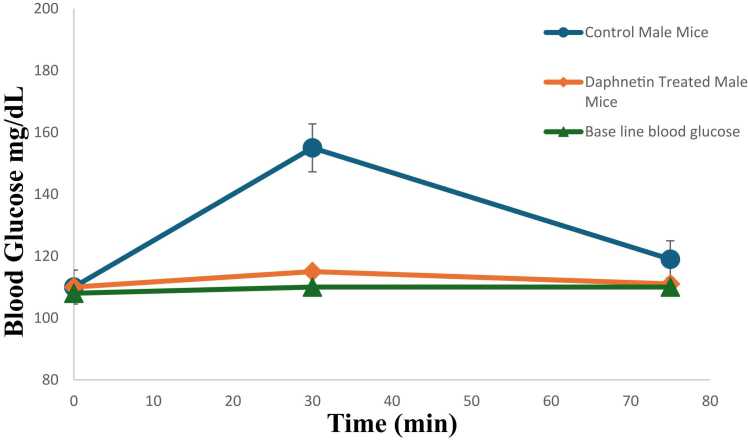

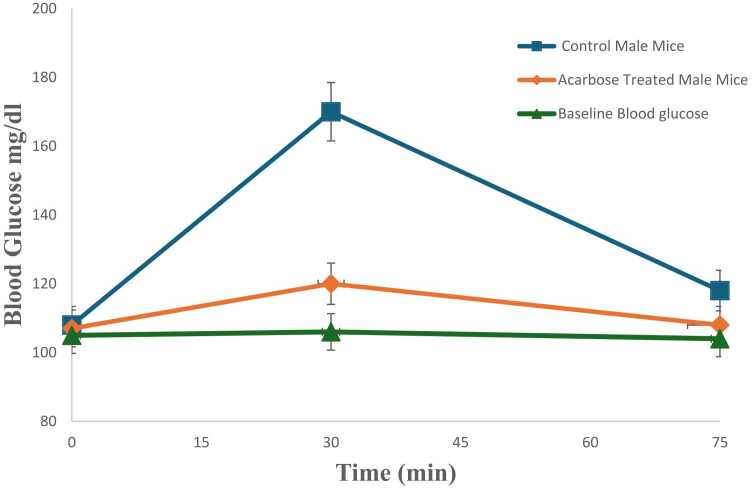
**A**. Blood sugar concentrations following OSTT in normal control mice and daphnetin treated mice. Error bars represent mean± SD and p values < 0.05 were considered significant. **B**. Blood sugar concentrations following OSTT in normal control mice and acarbose treated mice. The values have been compared with fasting (baseline) blood glucose level. Error bars represent mean± SD of triplicates and p values < 0.05 were considered significant.

OSTT results have been shown in Figs. 4A and 4B. Prior treatment with daphnetin prevented the post meal glucose spike in OSTT and the effect was comparable to acarbose. (Fig. 4A, B)

### 3.6 Estimation of serum enzymes

**Fig. 5:**
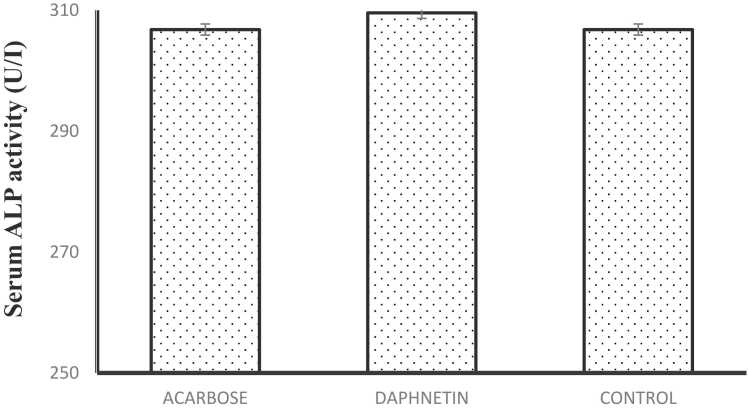

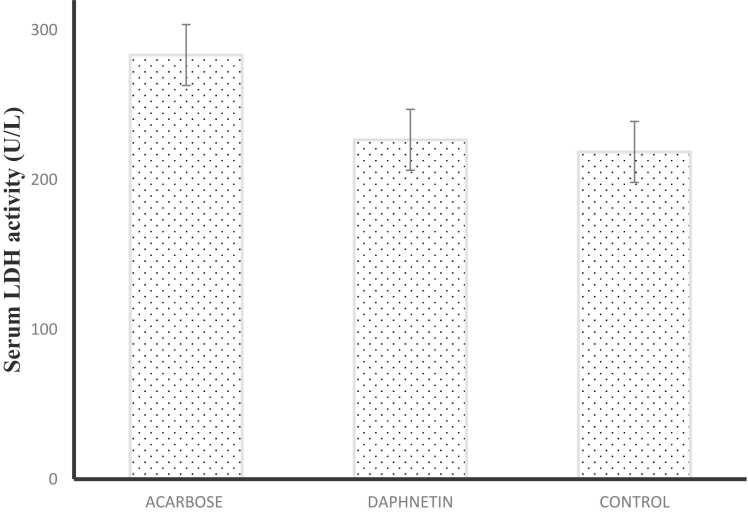
**A:** Serum ALP activity in normal, daphnetin and acarbose treated mice serum. Error bars represent mean± SD of triplicates. **5B:** Serum LDH activity in normal, daphnetin and acarbose treated mice serum. Error bars represent mean± SD of triplicates.

The serum ALP level remained unchanged in daphnetin and acarbose treated mice compared to normal mice. Serum LDH level was slightly increased in acarbose treated mice serum while the level was almost same in both normal and daphnetin treated mouse serum (Fig. 5A, B) This result indicates that daphnetin had no toxic effect on the liver or cellular toxicity.

### 3.7 Histological studies

**Fig. 6:**
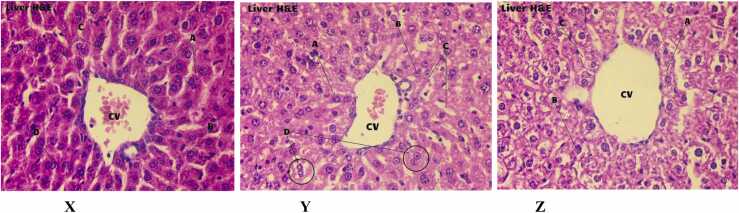

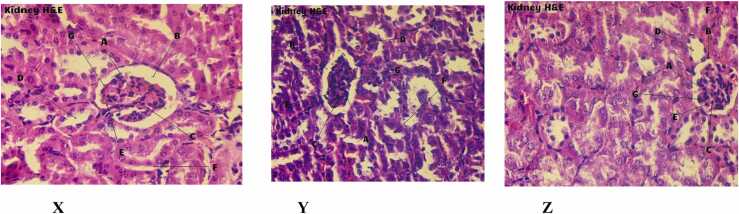

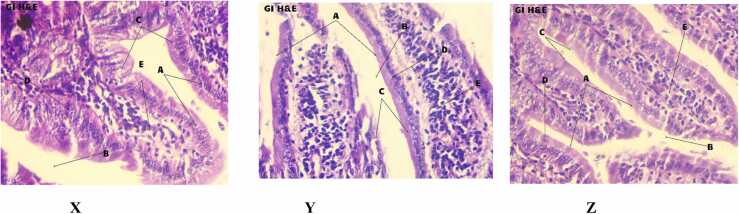
**A:** H&E staining of liver sections collected from normal mouse (X) and mouse treated with Daph (Y), Acarbose (Z) (magnification 400X). **A**- Sheets of Hepatocytes, **B**- Prominent Nucleoli, **C**- Sinusoids, **D**- Binucleate hepatocytes. **CV –** Central Vein. Fig. 6**B:** H&E staining of kidney sections collected from normal mouse (X) and mouse treated with Daph (Y), Acarbose (Z) (magnification 400X). **A**- Glomerulus **B**- Glomerular Capsular Space, **C**- Extra glomerular mesangial Cell, **D**- Distal convoluted tubule, **E**- Podocytes, **F**- Proximal convoluted tubule, **G**- Glomerular capsule or Bowman's capsule. Fig. 6**C:** H&E staining of sections of small intestine collected from normal mice (X) and mice treated with Daph (Y), Acarbose (Z) (magnification 400X). **A**- Intestinal Villi, **B**- Lumen, **C**- Absorptive Epithelium, **D**- Goblet Cell, **E**- Lamina Propria.

The physiological patterns of different portions of liver collected from normal, daphnetin and acarbose treated mice have been demonstrated in Fig. 6A. The hepatocytes were in prominent form, no damage had occurred in the central vein, nucleoli and sinusoids in daphnetin and acarbose treated mice. All the physiological structures remained the same as in the control mice (viewed in 400X magnification).

The H&E staining of kidney tissues revealed no occurrence of abnormalities due to daphnetin and acarbose treatment. No sclerosing and necrotizing zones have been found. The podocytes and tubular parts also appeared in the prominent form (Fig. 6B).

The small intestinal tissues were viewed with the help of 400X magnifications as demonstrated in the Fig. 6C. The cellular pattern and structures of the selected zones of intestinal tissues in daphnetin and acarbose treated mice were recognised as normal and were the same as in the control mice. No atrophy, damage or lymphocytic infiltration of lamina propria was found. From these observations, it can be said that daphnetin/acarbose administration didn’t cause Celiac disease. There was no increased capillary angiogenesis, mucosal edema. Also, there was no decrease in total absorptive surface area.

### 3.8 Free radical scavenging activity

**Fig. 7:**
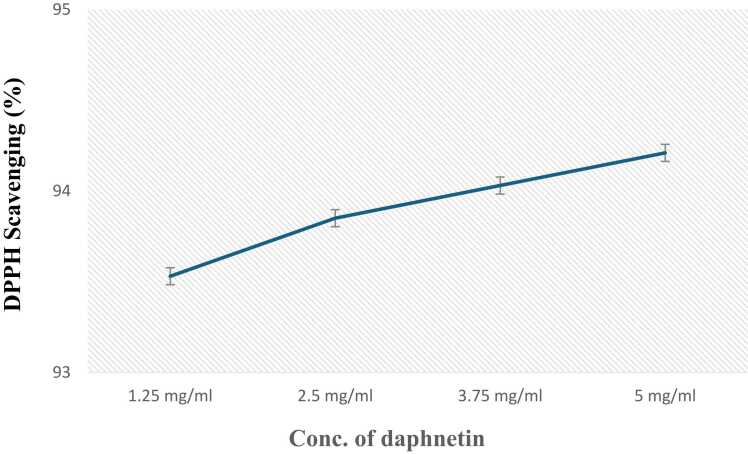
Dose dependent DPPH scavenging activity of daphnetin. Error bars represent mean± SD of triplicates and p values < 0.05 were considered significant.

Daphnetin was found to act as an effective antioxidant molecule. The free radical scavenging activity was found to increase with increase in the concentration of daphnetin. (Fig. 7)

## 4 Discussion

DHCs are known natural compounds and possess several beneficial roles including free radical scavenging activity. Here, we have evaluated the anti α-amylase activity of daphnetin *in vitro* and *in vivo*. Daphnetin has been found to inhibit HSA and PPA potentially while it has only 50 % inhibitory role against AG *in vitro*. On the other hand, acarbose was found to inhibit HSA and AG completely but had only 50 % activity against AG. The intrinsic fluorescence intensity of all the enzymes decreased when mixed with daphnetin in increasing concentrations. The fluorescence spectrum is associated with the polarity of tryptophan and tyrosine residue microenvironment. Thus, fluorescence quenching of the enzymes by daphnetin may be because of the interactions between tyrosine/tryptophan residues and the inhibitor which lead to changes in the microenvironment of the enzymes. The Stern-Volmer association constant value (Ka), which is also a measure of the affinity of the enzyme for the ligand, remains almost the same at the tested temperatures (301 K, 310 K and 323 K), which indicates the stability of the enzyme inhibitor complex. The ΔG° value calculated from the binding constant (between HSA and daphnetin) was negative, with a maximum ΔG° value of −21.55 kJ /mol at 301 K, indicating favourable interactions occurring between the enzyme and the inhibitor.

The *in vivo* anti amylase activity of daphnetin was confirmed by OSTT which showed significant reduction in blood glucose level in daphnetin treated mice prior to starch administration. Administration of daphnetin had no effect on the blood serum markers like LDH, ALP and on histology of organs like liver, kidney and small intestine. The effect of daphnetin was comparable to that of positive control, acarbose.

Daphnetin is found in many plants and used as an anti-inflammatory molecule for experimental purposes [42], [43]. It also acts as an immunosuppressive and mediates its action through the suppression of signalling pathways in the T cells in mice [44]. It is known that daphnetin alleviates several conditions like pathological oedema, infiltration of neutrophils and inflammation, vacuolization, necrosis, cellular apoptosis in rats with severe acute pancreatitis at a dose of 4 mg/kg, intraperitoneally. The LD50 value of daphnetin is too high, 5370 mg/kg when given orally in mice [45]. It satisfies Lipinski rule of 5 and doesn’t cross the blood brain barrier. Moreover, a recent study shows that pretreatment with daphnetin has a protective role in INS-1 (insulinoma) pancreatic β-cells against streptozotocin-induced apoptosis [46].

Thus, daphnetin or derivatives of it might be used for dual purpose, blocking α-amylase in the gut and stimulating insulin secretion in blood and subsequent regulation of the apoptotic pathway. Hence, careful investigation should be conducted on daphnetin to reveal its potential benefits.

## 5 Conclusion

Daphnetin or 7,8 dihydroxycoumarin was found to be a potential salivary and pancreatic α-amylase blocker both *in vivo* and *in vitro*. It also inhibited α-glucosidase but to a lesser extent. Application of daphnetin for 7 days had no toxic effect on the organs like liver, kidney, small intestine in mice. Hence, it can be concluded that daphnetin has the potential to act as an α-amylase blocker. Further studies and careful investigations can lead to a more conclusive role of daphnetin in future.

## Funding

The work was funded by the Intramural research fund (IMPSXC2022-23/002) provided by St. Xavier’s college, Kolkata, India.

## CRediT authorship contribution statement

**Sarkar Kasturi:** Writing – review & editing, Supervision, Project administration, Investigation, Conceptualization. **Saha Shraddha:** Writing – original draft, Investigation. **Dutta Sagnik:** Writing – original draft, Investigation. **Kar Sayan:** Writing – original draft, Methodology, Investigation, Data curation. **Sil Parames:** Writing – review & editing, Supervision. **Giri Nabanita:** Resources. **Chatterjee Shreya:** Resources.

## Declaration of Competing Interest

The authors declare that they have no known competing financial interests or personal relationships that could have appeared to influence the work reported in this paper.

## Data Availability

Data will be made available on request.

## Glossary

AG: α-glucosidase
ALP: Alkaline phosphatase
DHCs: Dihydroxycoumarins
DPPH: 1,1-diphenyl-1-picrylhydrazyl
F/F0: fluorescence intensity ratio
LD50: Lethal dose 50
LDH: Lactate dehydrogenase
ΔG°: Standard Gibbs free energy change
GI: Gastrointestinal tract
H&E: Haematoxylin and Eosin
HSA: Human salivary amylase
IC50: Inhibitory concentration 50
K_a_: association constant
Km: Michaelis constant
Ksv: Stern Volmer constant
O.D: Optical density
OSTT: Oral starch tolerance test
PP: Postprandial
PPA: Pancreatic α-amylase
R^2^: The coefficient of determination
Vmax: Velocity maximum
